# Promotion of ubiquitination-dependent survivin destruction contributes to xanthohumol-mediated tumor suppression and overcomes radioresistance in human oral squamous cell carcinoma

**DOI:** 10.1186/s13046-020-01593-z

**Published:** 2020-05-14

**Authors:** Ming Li, Feng Gao, Xinfang Yu, Qing Zhao, Li Zhou, Wenbin Liu, Wei Li

**Affiliations:** 1 Cell Transplantation and Gene Therapy Institute, The Third Xiangya Hospital, Central South University, Changsha, Hunan 410013 People’s Republic of China; 2 Changsha Stomatological Hospital, Changsha, Hunan 410004 People’s Republic of China; 3 School of Stomatology, Hunan University of Chinese Medicine, Changsha, Hunan 410208 People’s Republic of China; 4 Xiangya Stomatological Hospital & School of Stomatology, Central South University, Changsha, Hunan 410000 People’s Republic of China; 5 Department of Ultrasonography, The Third Xiangya Hospital of Central South University, Changsha, Hunan 410013 People’s Republic of China; 6 Department of Pathology, Xiangya Hospital of Central South University, Changsha, Hunan 410008 People’s Republic of China; 7 Department of Pathology, Hunan Cancer Hospital, Changsha, Hunan 410013 People’s Republic of China; 8 Department of Radiology, The Third Xiangya Hospital of Central South University, Changsha, Hunan 410013 People’s Republic of China

**Keywords:** Oral squamous cell carcinoma, Xanthohumol, Ubiquitination, Fbxl7

## Abstract

**Background:**

Overexpression of survivin plays a crucial role in tumorigenesis and correlates with poor prognosis in human malignancies. Thus, survivin has been proposed as an attractive target for new anti-tumor interventions.

**Methods:**

A natural product library was used for natural compound screening through MTS assay. The expression of survivin in oral squamous cell carcinoma (OSCC) and the inhibitory effect of xanthohumol (XN) on OSCC were examined by anchorage-dependent and -independent growth assays, immunoblot, immunofluorescence, immunohistochemical staining, ubiquitination analysis, co-immunoprecipitation assay, CRISPR-Cas9-based gene knockout, and xenograft experiment.

**Results:**

Survivin is highly expressed in OSCC patient-derived tissues and cell lines. Knockout of survivin reduced the tumorigenic properties of OSCC cells in vitro and in vivo. With a natural compound screening, we identified that xanthohumol inhibited OSCC cells by reducing survivin protein level and activating mitochondrial apoptotic signaling. Xanthohumol inhibited the Akt-Wee1-CDK1 signaling, which in turn decreased survivin phosphorylation on Thr34, and facilitated E3 ligase Fbxl7-mediated survivin ubiquitination and degradation. Xanthohumol alone or in combination with radiation overcame radioresistance in OSCC xenograft tumors.

**Conclusion:**

Our findings indicate that targeting survivin for degradation might a promising strategy for OSCC treatment.

## Background

Oral cancer is one of the most common cancers worldwide, accounting for 2% of all cancer cases. Over 90% of oral malignancies were diagnosed as oral squamous cell carcinoma (OSCC), which arise from the epithelial mucosa of the oral cavity [1–3]. Beyond genetic susceptibility caused by gene mutation, multiple environmental factors have been considered to contribute to the initiation of OSCC [4]. Tobacco, alcohol, and betel nut chewing remain the major culprits with a well-established synergistic effect for the tumorigenesis of OSCC. The highest incidence of OSCC is observed in South Asian countries and regions, such as India, Sri Lanka, and the south-central of China, which are caused by the high rates of cigarette smoking and areca nut use in these areas [5–7]. Although the improvements in early diagnosis and surgical treatment over the past decades, OSCC usually progresses rapidly and presents high mortality rates, especially in patients with an advanced stage, and no targeted therapy is used in clinic for OSCC treatment currently [3, 6–9]. Thus, a better understanding of the mechanisms of OSCC oncogenesis and development of novel anti-tumor targets and drugs, are still the urgent demand for OSCC treatment.

Survivin is an evolutionarily conserved eukaryotic protein that plays a critical role in cell cycle G2/M transition and apoptosis inhibition [10]. Together with the Aurora B kinase, survivin constitutes an integral component of chromosomal passenger complex (CPC) which guarantees proper chromosomes and segregation during mitosis [10, 11]. Beyond the key role in cell division, as a member of the inhibitor of apoptosis protein family (IAPs), survivin is frequently overexpressed in human cancers and confers the unlimited cell proliferation and apoptosis resistance [11–14]. Previous reported showed that survivin is predominantly localized in the cytoplasm of cancer cells and is generally believed to function as apoptotic suppressor to maintain survival of cancer cells. In contrast, the smaller fraction of nuclear localized survivin is considered to act as a component of CPC complex to promote cell cycle progression, suggesting that the different subcellular pools of survivin exhibit distinct biological functions [13–16]. Thus, as an oncoprotein that is both essential for mitosis and apoptosis inhibition, survivin seemed a promising novel target for anti-cancer treatment. Although overexpression of survivin is positively correlated with poor prognosis in multiple human cancers, the function of survivin in human OSCC remains undefined.

In this study, we investigate the biological function of survivin in human OSCC cells. With a compound screening, we identified the natural product, xanthohumol, as a potential survivin inhibitor for OSCC treatment. We determined the anti-tumor effect of xanthohumol in OSCC cells and elucidate the underlying mechanisms using the in vitro and in vivo models.

## Materials and methods

### Reagents and cell culture

Chemical reagents, including DMSO, SDS, NaCl, and Tris base, were purchased from Sigma-Aldrich (St. Louis, MO). MG132 and cycloheximide (CHX) were obtained from Thermo Fisher Scientific (Waltham, MA). The DMEM, RPMI-1640 medium, and Fetal Bovine Serum (FBS) for cell culture were purchased from Invitrogen (Grand Island, NY). Human oral squamous cell carcinoma (OSCC) cells, including CAL27, SCC15, SCC25, and SCC9 were purchased from American Type Culture Collection (ATCC, Manassas, VA). The cells were maintained according to ATCC protocols in a 37 °C humidified incubator with 5% CO_2_. The ionizing radiation acquired resistance cell lines CAL27-IR and SCC25-IR were established in our laboratory by exposing CAL27 and SCC25 cells to gradually increasing dose of ionizing radiation for approximately 6 months. Briefly, CAL27 and SCC25 cells were serially irradiated with 2Gy of X-rays to a final dose of 80 Gy. Cells were cultured with DMEM medium and maintained at a 37 °C humidified incubator with 5% CO_2_. The irradiated cells were passaged into new culture flask when growing to approximately 80% confluence. Reirradiation of Xrays was repeated over a period of 6 months (starting at 2 Gy and ending with 8 Gy for each irradiation, the dose was gradually increased for each month by 2 Gy), for a total dose of 80 Gy. Immortalized oral epithelial cell hTERT-OME was purchased from Applied Biological Materials (ABM) Inc. (Richmond, BC, Canada). The HaCat cells were purchased form Thermo Fisher Scientific (Waltham, MA). All cells were subjected to mycoplasma analysis every 2 months. Primary antibodies against Survivin (#2808), cleaved-PARP (#9532), VDAC1 (#4661), Bax (#14796), cleaved-caspase 3 (#9664), cytochrome C (#11940), ubiquitin (#43124), β-actin (#3700), ubiquitin (#3936), p-Survivin Thr34 (#8888), α-tubulin (#3873), p-Akt Ser473 (#4060), p-Wee1 Ser642 (#4910), His-tag (#12698), HA-tag (#2367), p-CDK1 Tyr15 (#4539), and p-CDK1 Thr161 (#9114) were obtained from Cell Signaling Technology, Inc. (Beverly, MA). Antibodies against Ki67 (ab16667) and FbxL7 (#ab59149) were purchased from Abcam (Cambridge, UK). Flag-tag (F3165) antibody was obtained from Sigma Aldrich (St. Louis, MO). Lipofectamine® 2000 (Thermo Fisher Scientific) was used for plasmid transfection following the manufacturer’s instructions.

### MTS assays

MTS assay was performed as described previously [17]. Briefly, human OSCC cells were seeded in 96-well plates at a concentration of 2 × 10^3^/well. Cells were treated with DMSO control or xanthohumol for various time points (0, 24, 48, and 72 h), cell viability was determined by MTS assay (G3581, Promega, Madison, WI).

### Anchorage-independent cell growth

The anchorage-independent cell growth assay was performed as described previously [18]. Briefly, the Eagle’s basal medium containing 10% FBS, 0.6% agar, and different concentration of xanthohumol was loading to a six-well plate as an agar base. Human OSCC cells were counted at the concentration of 8000 cells/ml and seeded into 6-well plates with 0.3% Basal Medium Eagle agar containing 10% FBS and xanthohumol. The cultures were maintained at 37 °C in a 5% CO_2_ incubator for 2 weeks and colonies were counted.

### Western blotting

Whole-cell extract (WCE) was prepared with RIPA buffer (#PI89901, Thermo Fisher Scientific) supplemented with protease inhibitors and concentrated by BCA protein assay. Western blotting was performed as previously described [19]. Briefly, A total of 30 μg WCE was mixed with loading buffer and boiled at 95 °C for 5 min, followed by SDS-PAGE electrophoresis and electrotransfer. The non-fat milk (5%) was used for membrane blocking at room temperature for 30 min and the membrane was incubated with the primary antibody at 4 °C overnight. After incubation with anti-rabbit/mouse IgG HRP second antibody, the target protein was visualized by chemiluminescence.

### Immunohistochemical staining (IHC)

The tissues from xenograft tumors were fixed in 10% neutral-buffered formalin and subjected to immunohistochemical staining as described previously [20]. First, the tissue slides were deparaffinized, followed by rehydration using various concentrations of ethanol. Antigen retrieval was performed by boiling the slides with the sodium citrate buffer (10 mM, pH 6.0) for 10 min. The slides were incubated with 3% H_2_O_2_ in methanol for 10 min to deactivate endogenous peroxidase. After washing with PBS, slides were blocked with 50% goat serum albumin in PBS for 1 h at room temperature, followed by incubation with primary antibody (4 °C, overnight) and second antibody (room temperature, 45 min) in a humidified chamber. The target protein was visualized by DAB substrate and hematoxylin was used for counterstaining.

### Natural compound screening

The 70 compounds of interest were selected from the Natural Product Library (Cat. No. L1400–01/02) from Selleck Chemicals (Houston, TX). CAL27 cells were seeded in a 96-well plate. After overnight incubation, cells were treated with 1 μM of DMSO (control) or natural compounds for 48 h. Cell viability was examined by the MTS assay. Tested compounds are listed in Supplementary Table 1.

### Generation of survivin knockout stable cell lines

Two different single-guide RNAs (sgRNAs) were used to generate CRISPR-Cas9-based survivin knockout constructs (sgSurvivin#1forward, 5′- CAGTTTGAAGAATTAACCCT-3′, reverse, 5′-AGGGTTAATTCTTCAAACTG-3′, sgSurvivin#2 forward, 5′- GAACATAAAAAGCATTCGTC-3′, reverse, 5′-GACGAATGCTTTTTATGTTC-3′). The sgSurvivin plasmid was transiently transfected to the OSCC cells. Puromycin (1 μg/mL) was added to cell culture medium and maintained for 3 weeks for signal clone selection. The Fbxl7 siRNA (sc-62,306), Akt1/2 siRNA (sc-43,609), and siCtrl (sc-37,007) were purchased from Santa Cruz Biotechnology (Dalla, TX). The primers for survivin qRT-PCR analysis is forward: CCACTGAGAACGAGCCAGACTT, reverse: GTATTACAGGCGTAAGCCACCG.

### Ubiquitination analysis

The Ubiquitination analysis was performed as described previously [21]. Briefly, for endogenous ubiquitination detection, cells were lysed with modified RIPA buffer (20 mM NAP, pH 7.4, 150 mM NaCl, 1% Triton, 0.5% Sodium-deoxycholate, and 1% SDS) supplemented with 10 mM N-Ethylmaleimide (NEM) and protease inhibitors. The lysates were sonicated for 30 s, boiled at 95 °C for 15 min, and diluted with 0.1% SDS containing RIPA buffer, then centrifuged at 16000×g for 15 min at 4 °C. The supernatant was subjected to IP with survivin antibody, survivin ubiquitination was determined by IB analysis. For Nickel pull-down assay, cell lysates were prepared with lysis buffer (6 M guanidine-HCl, 0.1 M Na2HPO4/NaH2PO4, 0.01 M Tris/HCl, pH 8.0, 5 mM imidazole, and 10mMβ-mercaptoethanol) supplemented with protease inhibitors and 10 mM N-Ethylmaleimide (NEM). The lysates were sonicated for 30 s, followed by incubation with 50 ml Ni-NTA-agarose (QIAGEN Inc., Valencia, CA) for 4 h at room temperature. The beads were washed with specific washing buffer and boiled with 2 × SDS loading buffer containing 200 mM imidazole and subjected to western blotting.

### Immunofluorescence (IF)

Cells (5 × 10^3^/well) seeded in a chamber slide were treated with vehicle control, XN (5 μM, 24 h), IR (4 Gy), or a XN and IR combination. Cells were maintained in the incubator for another 72 h. Cells were fixed with 4% paraformaldehyde and permeabilized in 0.5% Triton X-100 for 20 min, followed by blocking in 5% BSA for 1 h and overnight incubation with γ-H2AX antibody at 4 °C in a humidified chamber. Alexa Fluor 488 dye-labeled anti-rabbit IgG was used as the secondary antibody. Nuclei were counterstained with DAPI (cat. #P36935, Thermo Fisher Scientific). The following antibodies were used for IF: γ-H2AX (cat. #ab11174, 1:100), goat anti-rabbit IgG Alexa Fluor 488 (cat. #ab150077, 1:500).

### Plate colony formation assay

The cells were treated with vehicle control, XN, IR, or a XN and IR combination and seeded into a 6-cm plate (400 cells/well). The cultures were maintained for 2 weeks at 37 °C in a 5% CO_2_ incubator. The colonies were fixed with 4% paraformaldehyde, stained with 0.5% crystal violet, and counted under a microscope. Three independent experiments were performed as indicated.

### In vivo tumor growth assay

The in vivo animal study was approved by the Institutional Animal Care and Use Committee (IACUC) of Central South University (Changsha, China). (2 × 10^6^) cells were s.c.injected into the 6-week-old athymic nude mice (*n* = 6) at the right flank to generate the xenograft mouse model. Compound treatment was initiated when tumor volume reached at 100 mm^3^. Xanthohumol (10 mg/kg) was administrated by i.p. injection every 2 days, whereas the control mice were treated with vehicle control. For irradiation treatment, the tumor bearing mice were randomly divided into four groups (*n* = 6): 1,vehicle control (0.5% dimethyl sulfoxide, 100 mL/every 2 days, i.p.); 2, local ionizing radiation (2 Gy/ twice per week, irradiated with X-rays using X-RAD 320, Precision X-ray, Inc.,); 3, Xanthohumol (10 mg/kg/ every 2 days, i.p.); 4, Xanthohumol (10 mg/kg/ every 2 days, i.p.) + local ionizing radiation (2 Gy/ twice per week). Tumor volume was recorded with and determined with the formula: length × width × width/2. Tumor mass was subjected to IHC staining.

### Blood analysis

The EDTA-coated tubes were used for Mouse blood collection by cardiac puncture. The red blood cells (RBC), hemoglobin (Hb), white blood cells (WBC), alanine aminotransferase (ALT), aspartate aminotransferase (AST), and blood urea nitrogen (BUN) were analyzed at the Laboratory of the Third Xiangya Hospital of Central South University (Changsha, Hunan, China).

### Statistical analysis

The statistical analysis was conducted with GraphPad Prism 5 (San Diego, CA). The Student’s t-test or one-way ANOVA was used to evaluate the difference between tested groups, and a probability value of *p* < 0.05 was used as the criterion for statistical significance. The experiment was performed triplicate, and all quantitative data are expressed as mean ± sd.

## Results

### Survivin is highly expressed in OSCC tissues and cancer cells

**Fig. 1:**
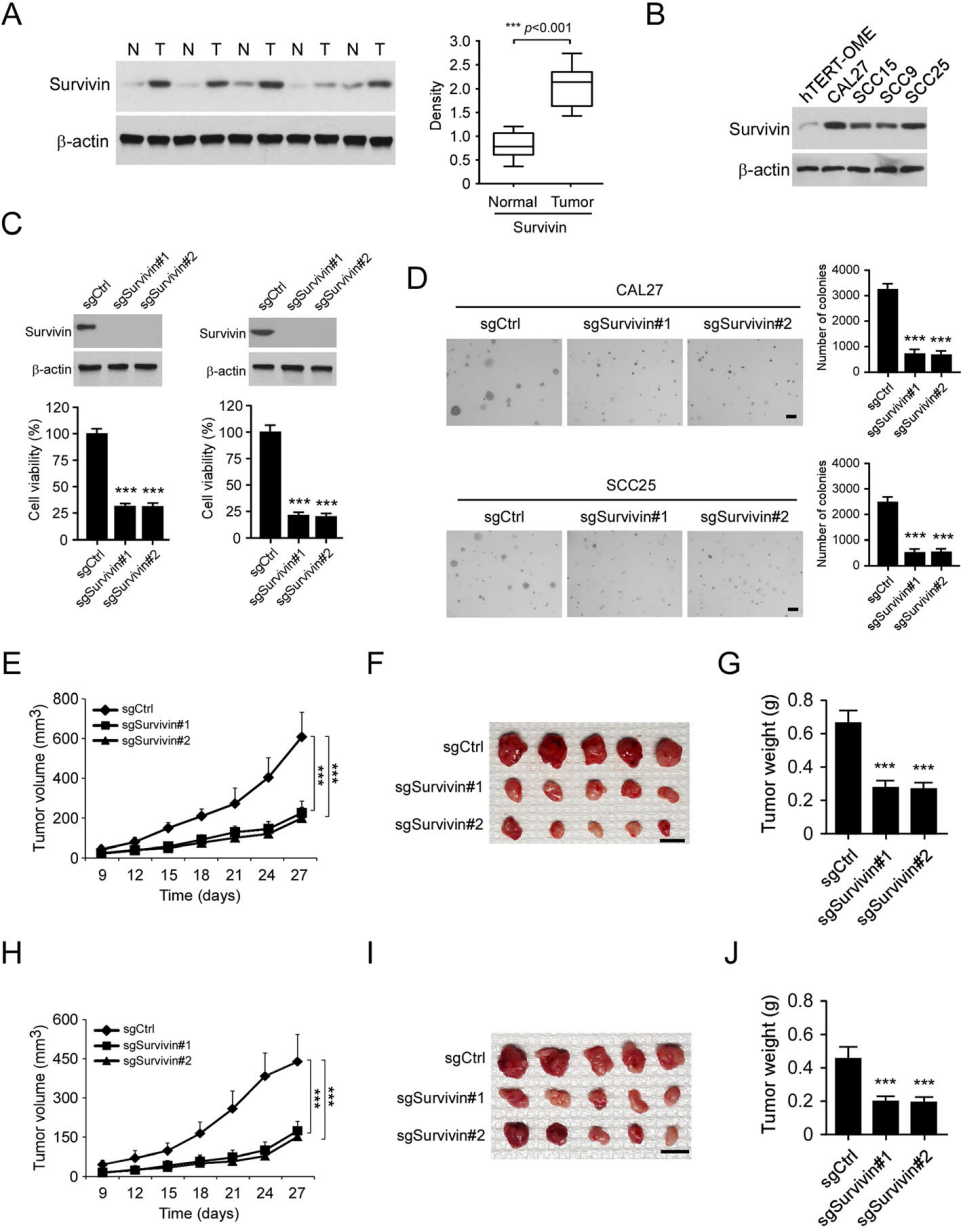
Overexpression of survivin is required for maintaining of tumorigenic properties of oral squamous cell carcinoma (OSCC) cells. **a** Left, the expression of survivin in five representative OSCC tissues was examined by IB analysis. A total of 35 cases of paired OSCC tissue and adjacent non-tumor tissue were subjected to IB analysis. Right, relative survivin expression was determined by quantification analysis. N, adjacent non-tumor tissue, T, tumor tissue. **b** The expression of survivin in immortalized oral epithelial cell and OSCC cells. **c** Knockout of survivin decreased cell viability in CAL27 (left) and SCC25 (right) cells. **d** Knockout of survivin inhibited colony formation of CAL27 (top) and SCC25 (bottom) cells. Scale bar, 200 μm. **e**-**g** The tumor volume (**e**), the image of tumor mass (**f**), and tumor weight (**g**) of CAL27-derived xenograft tumors with survivin knockout or not. **h**-**j** The tumor volume (**h**), image of tumor mass (**i**), and tumor weight (**j**) of SCC25-derived xenograft tumors with survivin knockout or not. ***p < 0.001. For **f** and **i**, Scale bar, 1 cm

To determine the role of survivin in human OSCC, we performed immunoblotting (IB) to examine the expression of survivin in human OSCC tissues. The result showed that the protein level of survivin is significantly upregulated in OSCC tissues when compared to the paired adjacent non-tumor tissues (Fig. 1a). Furthermore, as compared to the immortalized oral epithelial cell, survivin was expressed at a relatively higher level in all tested OSCC cell lines (Fig. 1b). These results indicate that high level of survivin might play a crucial role in the oncogenesis of OSCC. Using the CRISPR-Cas9 based gene knockout technology, we constructed survivin stable knockout CAL27 and SCC25 cells. The MTS data revealed that knockout of survivin inhibited cell viability significantly in CAL27 and SCC25 cells (Fig. 1c). Moreover, the colony formation of survivin depletion OSCC cells was decreased robustly (Fig. 1d), suggesting that survivin deficiency attenuated both anchorage-dependent and -independent cell growth of OSCC cells. We next used a xenograft tumor model to determine the effect of survivin on in vivo tumor development of OSCC cells. Our data revealed that depletion of survivin caused a significant reduction of tumor volume and tumor weight in CAL27-derived xenograft tumors (Fig. 1e-g). Consistently, a similar inhibitory effect was observed in SCC25 xenograft tumors (Fig. 1h-j). These results support the notion that survivin is overexpressed in OSCC tissues and cell lines, and that knockout of survivin reduces the tumorigenic properties of OSCC cells.

### Xanthohumol is a candidate compound for OSCC inhibition

**Fig. 2:**
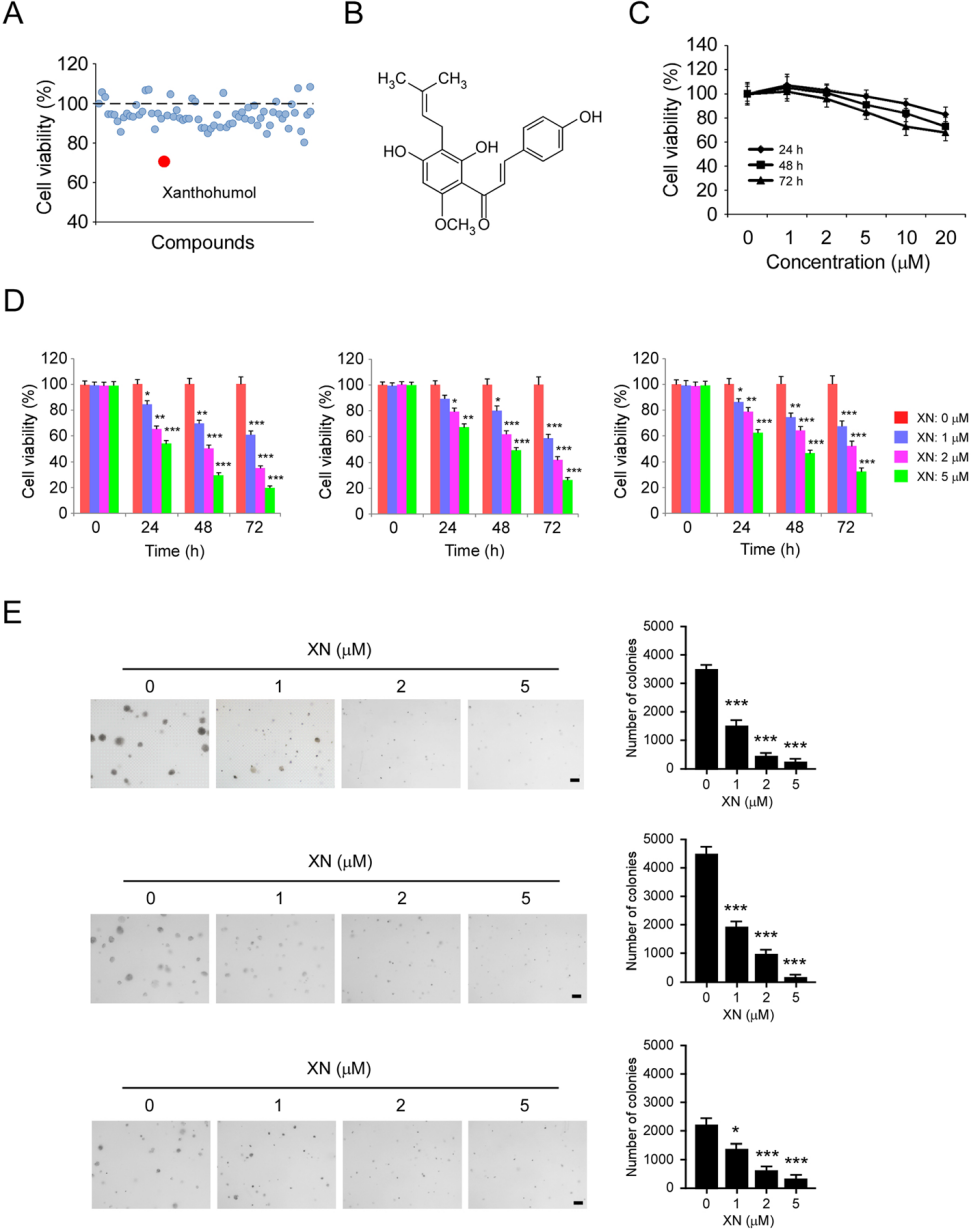
Xanthohumol (XN) suppresses OSCC cells. **a** Xanthohumol is a candidate natural product which inhibits OSCC cell viability significantly. A total of 70 compounds (1 μM) of interest were treated with CAL27 cells for 48 h. Cell viability was examined by the MTS assay. **b** The chemical structure of xanthohumol. **c** MTS assay analysis of the effect of xanthohumol on immortalized HaCat cells. **d** The effect of xanthohumol on cell viability of OSCC cells. SCC25 (left), SCC9 (middle), and CAL27 (right) cells were treated with xanthohumol or DMSO control. Cell viability was examined by the MTS assay. **e** The effect of xanthohumol on colony formation of OSCC cells. SCC25 (top), SCC9 (middle), and CAL27 (bottom) cells were treated with xanthohumol or DMSO control, the colony formation efficacy of OSCC cells was determined by soft agar assay. XN, xanthohumol. *p < 0.05, **p < 0.01, ***p < 0.001. Scale bar, 200 μm

To discover natural products (Supplementary Table 1) that can inhibit survivin and OSCC, we screened a natural compound library containing 70 compounds of interest by MTS assay. Our data showed that only xanthohumol caused a reduction of cell viability in CAL27 cells by over 30% at a single dose of 1 μM (Fig. 2a and b). We therefore focused on xanthohumol for further study. To determine the toxicity of xanthohumol on normal cells, we treated the immortalized human keratinocyte cell line HaCat for various time points. The result revealed that 72 h xanthohumol treatment did not cause a significant decrease in cell viability of HaCaT cells at the concentration less than 10 μM (Fig. 2c), indicating that xanthohumol is well-tolerated in immortalized non-tumor cells. We next validated the inhibitory effect of xanthohumol on a panel of human OSCC cells. Xanthohumol inhibited the cell viability of SCC25, SCC9, and CAL27 cells time- and dose-dependently (Fig. 2d). Treatment with 5 μM xanthohumol for 72 h caused a reduction of cell viability over 70% in all of these examined cells (Fig. 2d). Furthermore, the anchorage-independent growth assay showed that xanthohumol suppressed colony formation of OSCC cells significantly, and 5 μM xanthohumol decreased the efficacy of colony formation over 90% (Fig. 2e). Taken together, these results indicate that xanthohumol is a candidate compound that exhibits significantly anti-tumor effect in OSCC cells.

### Xanthohumol downregulates survivin expression and causes mitochondrial apoptotic signaling in OSCC cells

**Fig. 3:**
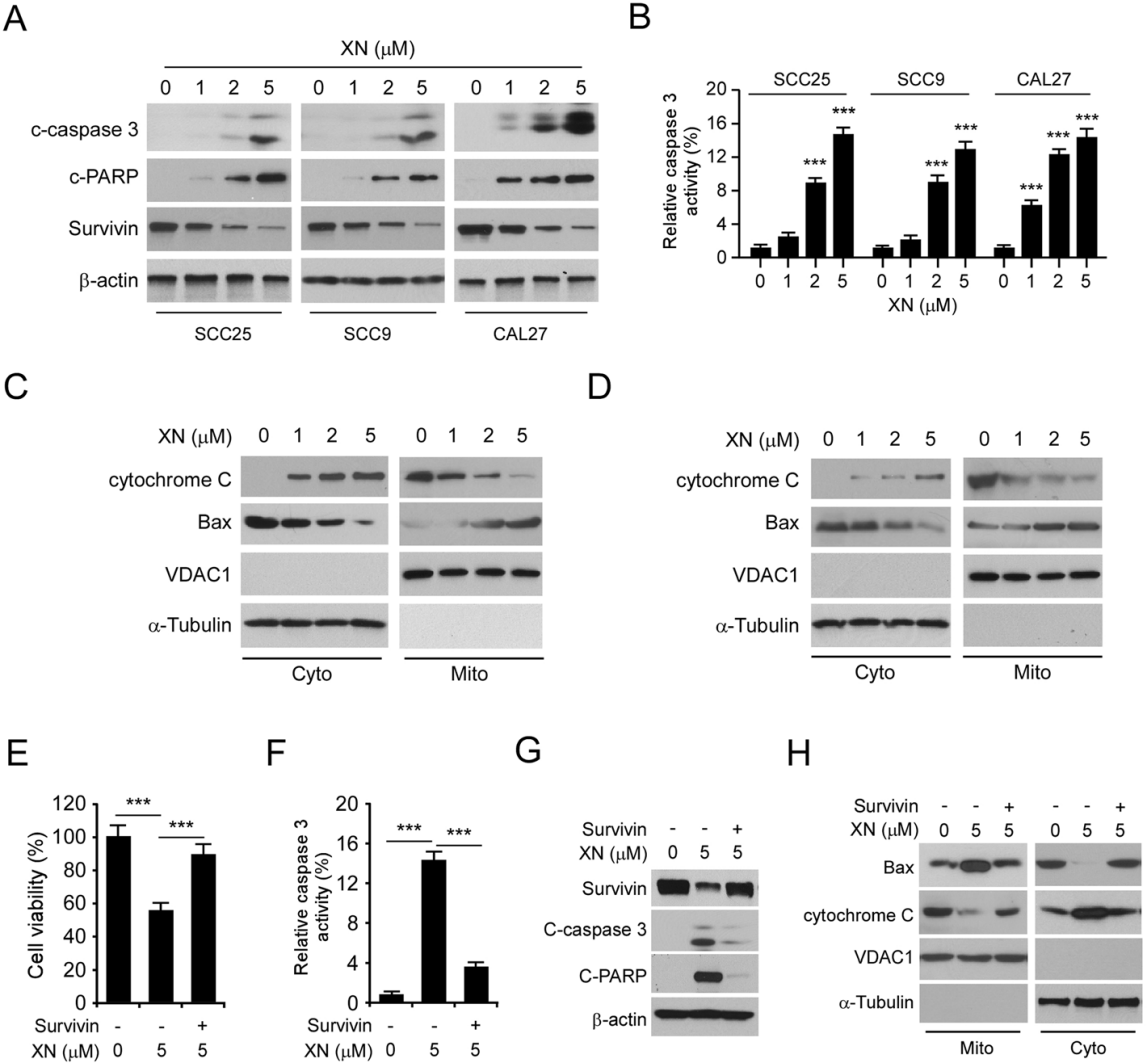
Xanthohumol causes mitochondrial apoptosis in OSCC cells through downregulating of survivin expression. **a** and **b** OSCC cells were treated with xanthohumol for 24 h, the whole-cell extract was subjected to IB analysis (**a**) and cleaved-caspase 3 activity measurement (**b**). **c** and **d** SCC25 (**c**) and CAL27 (**d**) cells were treated with xanthohumol for 24 h. Subcellular fractions were isolated and subjected to IB analysis. Cyto, cytosolic fraction; Mito, mitochondrial fraction. **e** Ectopic overexpression of survivin compromised xanthohumol-induced cell viability reduction. SCC25 cells were transfected with survivin cDNA and treated with xanthohumol for 24, cell viability was determined by MTS assay. **f** SCC25 cells were treated as in “Figure 3e”, whole-cell lysate was subjected to cleaved-caspase 3 activity analysis. **g** SCC25 cells were treated as in “Figure 3e”, whole-cell lysate was subjected to IB analysis. **h** SCC25 cells were treated as in “Figure 3e”, subcellular fractions were isolated and subjected to IB analysis. ***p < 0.001

To determine whether the anti-tumor effect of xanthohumol on OSCC cells is dependent on survivin downregulation, we examined survivin expression in xanthohumol-treated OSCC cells. The IB data showed that xanthohumol decreased the protein level of survivin in SCC25, SCC9, and CAL27 cells in a dose dependent manner (Fig. 3a). Moreover, cleaved-PARP and -caspase 3 increased substantially, indicating activation of apoptosis (Fig. 3a). Indeed, treatment with xanthohumol-upregulated the activity of caspase 3 (Fig. 3b). By isolation of the subcellular fractions from SCC25 (Fig. 3c) and CAL27 (Fig. 3d), we found that xanthohumol dose-dependently promoted the protein level of cytochrome C in the cytosolic fraction, whereas the expression of cytochrome C in mitochondrial fraction was reduced consistently (Fig. 3c and d). Furthermore, xanthohumol increased the presence of Bax on mitochondria and decreased it in the cytoplasm (Fig. 3c and d). This evidence indicates that xanthohumol activates the intrinsic apoptosis signaling. We next determined whether overexpression of survivin compromised xanthohumol-induced apoptosis. Our results revealed that ectopic overexpression of survivin compromised xanthohumol-induced decrease of cell viability in SCC25 (Fig. 3e) and CAL27 (Supplementary Figure 1A) cells. Consistently, the activity of cleaved-caspase 3 was reduced with survivin transfection (Fig. 3f, Supplementary Figure 1B). In addition, the overexpression of survivin attenuated xanthohumol-induced upregulation of cleaved-caspase 3, −PARP (Fig. 3g, Supplementary Figure 1C), and mitochondrial apoptosis (Fig. 3h, Supplementary Figure 1D). These data suggest that downregulation of survivin is required for xanthohumol-induced intrinsic apoptosis in OSCC cells.

### Xanthohumol promotes Fbxl7-mediated survivin ubiquitination and degradation

**Fig. 4:**
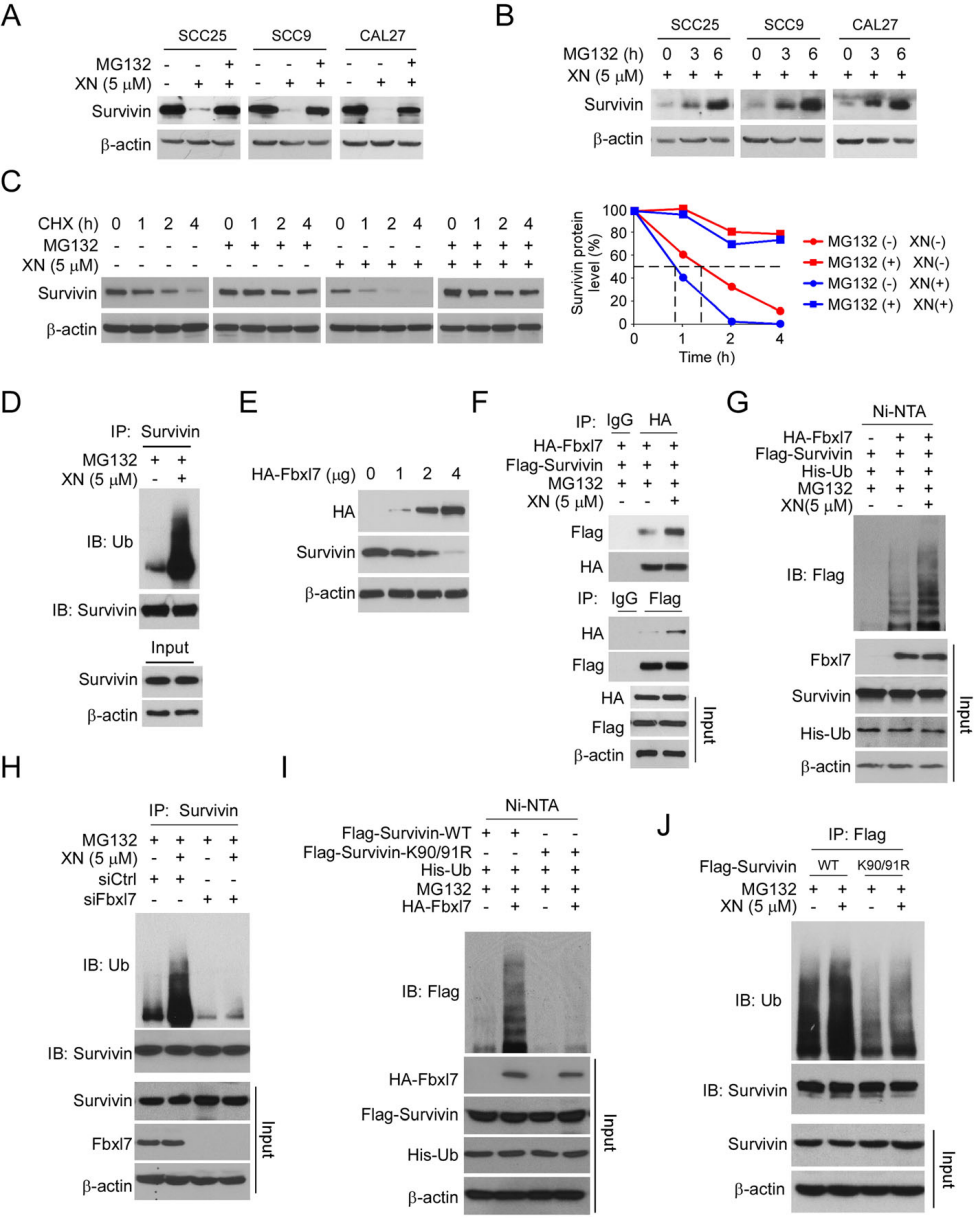
Xanthohumol promotes survivin ubiquitination and degradation in an Fbxl7-dependent manner. **a** MG132 rescued xanthohumol-induced survivin downregulation. OSCC cells were treated with xanthohumol, followed by MG132 treated for 6 h. The whole-cell extract was subjected to IB analysis. **b** MG132 rescued xanthohumol-induced survivin downregulation time-dependently. OSCC cells were treated with xanthohumol, followed by MG132 treated for various time points, whole-cell extract was subjected to IB analysis. **c** Xanthohumol shortened the half-life of survivin. SCC25 cells were treated with xanthohumol or DMSO control, followed by MG132 treated for 6 h or not, whole-cell extract was subjected to IB analysis. **d** Xanthohumol promoted survivin ubiquitination. SCC25 cells were treated with xanthohumol and subjected to ubiquitination analysis. **e** SCC25 cells were transfected with HA-Fbxl7 for 48 h. Cell lysates were subjected to IB analysis. **f** SCC25 cells were transfected with HA-Fbxl7 and Flag-Survivin plasmids as indicated, followed by xanthohumol treated for 24 h, the whole-cell lysate was prepared and subjected to Co-IP and IB analysis. **g** SCC25 cells were transfected with various plasmids as indicated, followed by xanthohumol treated for 24 h, the whole-cell lysate was subjected to ubiquitination analysis. **h** SCC25 cells were transfected with various siRNA as indicated, followed by xanthohumol treated for 24 h, the whole-cell lysate was prepared and subjected to Co-IP and IB analysis. **i** SCC25 cells were transfected with Flag-Survivin-WT or K90/91R mutant together with HA-Fbxl7 as indicated, followed by xanthohumol treated for 24 h, the whole-cell lysate was prepared and subjected to ubiquitination analysis. **j** SCC25 cells were transfected with Flag-Survivin-WT or K90/91R mutant and treated with xanthohumol for 24 h. The whole-cell lysate was prepared and subjected to ubiquitination analysis

To determine the mechanism of how xanthohumol downregulates survivin expression, we first performed qRT-PCR to analyze the transcription of survivin after xanthohumol treatment. The result showed that the mRNA level of survivin was unaffected in xanthohumol-treated OSCC cells (Supplementary Figure 2). Importantly, the proteasome inhibitor, MG132, restored survivin expression (Fig. 4a and b). Further study showed that xanthohumol shortened survivin half-life from 1.5 h to 45 min. Moreover, treated with MG132 extended the half-life over 4 h with either xanthohumol treated or not (Fig. 4c). These data suggest that xanthohumol decreased survivin expression is related to protein degradation. Indeed, the ubiquitination analysis revealed that treated with xanthohumol increased survivin ubiquitination in SCC25 cells (Fig. 4d). The previous report demonstrated that Fbxl7 is an E3 ligase which required for survivin degradation [22]. The IB result showed that ectopic overexpression of Fbxl7 reduced survivin protein level dose-dependently in OSCC cells (Fig. 4e). Moreover, the Co-IP results showed that xanthohumol enhanced the interaction between survivin and E3 ligase Fbxl7 (Fig. 4f). To further validate survivin ubiquitination in OSCC cells we co-transfected survivin and Fbxl7 in SCC25 cells. The results revealed that Fbxl7 promoted survivin ubiquitination robustly, and treatment with xanthohumol enhanced this process (Fig. 4g). Consistently, knockdown of Fbxl7 by siRNA impaired xanthohumol-induced survivin ubiquitination in OSCC cells (Fig. 4h), indicating that the E3 ligase Fbxl7 is required for xanthohumol-promoted survivin ubiquitination. The lysine residues (K) 90 and 91 are two ubiquitination sites required for ubiquitin ligation [22]. The ubiquitination analysis showed that double mutation of K90/91 substantially decreased xanthohumol-induced survivin ubiquitination when compare to survivin wild type (Fig. 4i). In addition, the survivin K90/91R mutant exhibited a robust reduction of ubiquitination either treated with xanthohumol or not (Fig. 4j). These results indicate that xanthohumol promotes survivin ubiquitination and degradation in an E3 ligase Fbxl7-dependent manner.

### Xanthohumol-decreased survivin Thr34 phosphorylation is required for survivin ubiquitination

**Fig. 5:**
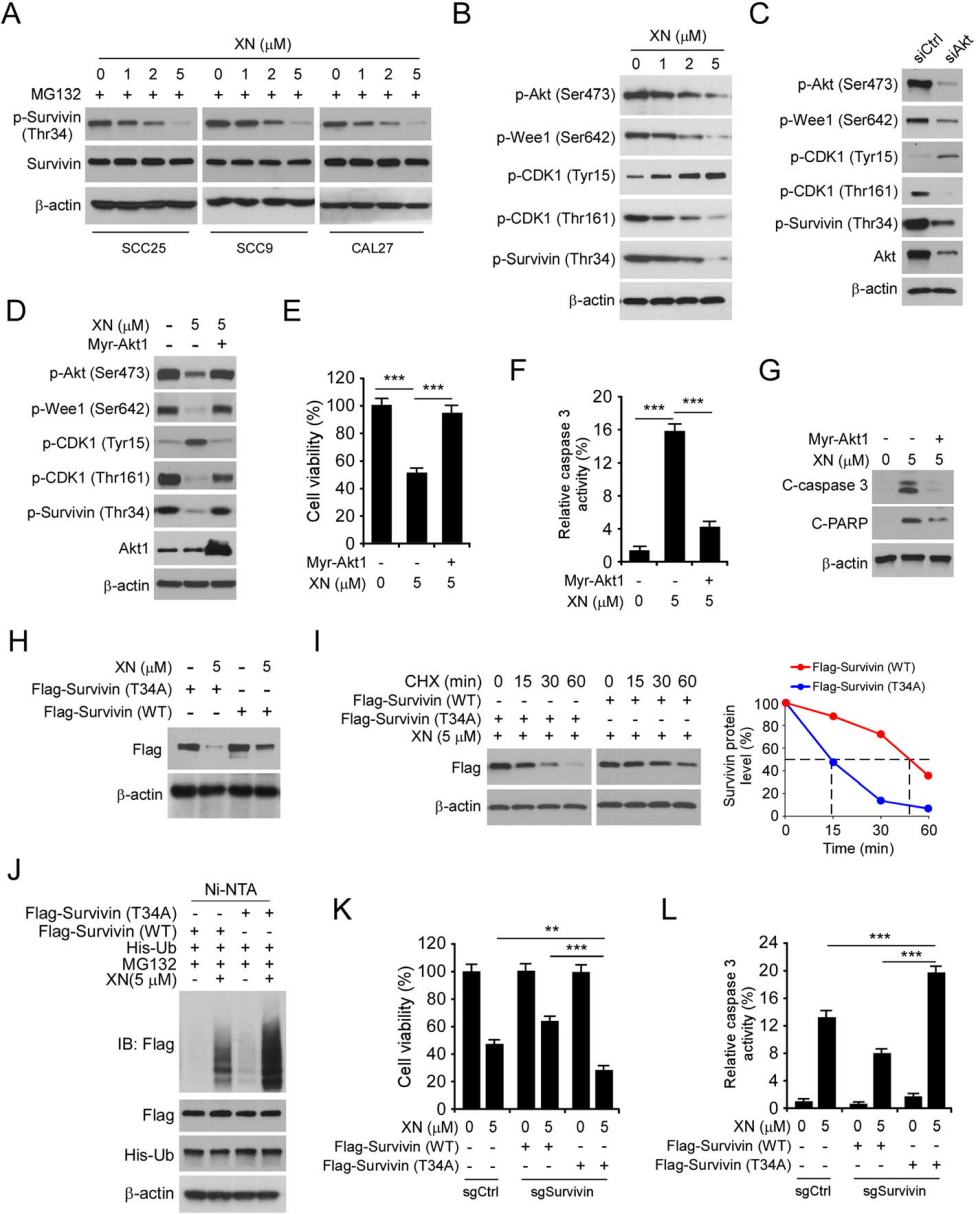
Xanthohumol-decreased survivin Thr34 phosphorylation is required for survivin ubiquitination. **a** and **b** OSCC cells were treated with xanthohumol for 24 h, the whole-cell lysate was subjected to IB analysis. **c** SCC25 cells were transfected with siCtrl or siAkt and treated with xanthohumol for 24 h, and whole cell lysate was subjected to IB analysis. **d** SCC25 cells were transfected with Mry-Akt1 and treated with xanthohumol for 24 h, the whole-cell lysate was subjected to IB analysis. **e**-**g** SCC25 cells were treated as in “Figure 5d”, cells were subject to MTS assay (**e**), cleaved-caspase 3 activity analysis (**f**), and IB analysis (**g**). **h** SCC25 cells were transfected with Flag-Survivin-WT or T34A and treated with xanthohumol for 24 h, the whole-cell lysate was subjected to IB analysis. **i** SCC25 cells were transfected with Flag-Survivin-WT or T34A and treated with xanthohumol for 24 h. CHX was added into the cell culture medium for various time points. The whole-cell lysate was subjected to IB analysis. **j** SCC25 cells were transfected with various plasmids and treated with xanthohumol for 24 h. The whole-cell lysate was subjected to ubiquitination analysis. **k** and **l** Survivin WT or knockout SCC25 cells were transfected with survivin WT or T34A mutant and treated with xanthohumol as indicated. Cells were subjected to MTS assay (**k**) and cleaved-caspase 3 activity analysis (**l**). ***p < 0.001

Because phosphorylation of survivin on Thr34 is required for the biological function and stability of survivin, we next determined whether xanthohumol regulates survivin phosphorylation. As shown in Fig. 5a, xanthohumol inhibited survivin Thr34 phosphorylation dose-dependently. Interestingly, the IB data revealed that xanthohumol decreased the activation of Akt signaling. The phosphorylation of Akt on Ser473 was inhibited with xanthohumol treatment (Fig. 5b). As the downstream target kinase of Akt, Wee1 phosphorylation was reduced coordinately (Fig. 5b). The previous study showed that Akt-dependent phosphorylation at Ser642 promotes the localization of Wee1 from nuclear to cytoplasmic, which is required for G2/M cell cycle arrest [23]. Our current data found that xanthohumol reduced Wee1 phosphorylation on Ser642, indicating that the nuclear xanthohumol promoted Wee1 nuclear localization. Indeed, Wee1-mediated CDK1 phosphorylation on Tyr15 was increased robustly with xanthohumol treatment. However, the phosphorylation of CDK1 on Thr161, which was a marker of CDK1 activation, was reduced dose-dependently (Fig. 5b). Moreover, knockdown of Akt by siRNA inhibited the phosphorylation of Wee1 (Ser642), CDK1 (Thr161), and survivin (Thr34), whereas the phosphorylation of CDK1 on Tyr15 was upregulated (Fig. 5c). Furthermore, ectopic overexpression of constitutively activated Akt, Myr-Akt1, rescued xanthohumol-induced reduction of phosphorylation of Wee1 (Ser642), CDK1 (Thr161), and survivin (Thr34) (Fig. 5d). Consistently, cell viability was increased in Myr-Akt1 transfected SCC25 cells (Fig. 5e). Additionally, the activity of cleaved-caspase 3 was attenuated significantly (Fig. 5f), and the protein level of cleaved-caspase 3 and -PARP were decreased (Fig. 5g) with Myr-Akt1 overexpression. These results suggest that inhibition of Akt signaling was required for xanthohumol-induced survivin Thr34 phosphorylation suppression and apoptosis induction.

We next constructed a survivin T34A mutant, in which the Thr 34 was mutated to Ala. The result showed that xanthohumol-induced a much stronger reduction of protein level of survivin T34A mutant (Fig. 5h). Moreover, the T34A mutation shortened the half-life of survivin from around 45 min to 15 min (Fig. 5i). We further compared xanthohumol-induced ubiquitination of survivin WT and T34A. The result showed that mutation of T34 to Ala increased survivin ubiquitination substantially after xanthohumol treatment (Fig. 5j). Strikingly, survivin WT, but not the T34A mutant, rescued cell viability (Fig. 5k) and decreased cleaved-caspase activity (Fig. 5l) in xanthohumol-treated survivin knockout cells. Collectively, our data suggest that the inhibition of phosphorylation is required for survivin ubiquitination and destruction in xanthohumol-treated OSCC cells.

### Xanthohumol suppresses in vivo tumor growth of OSCC cells

**Fig. 6:**
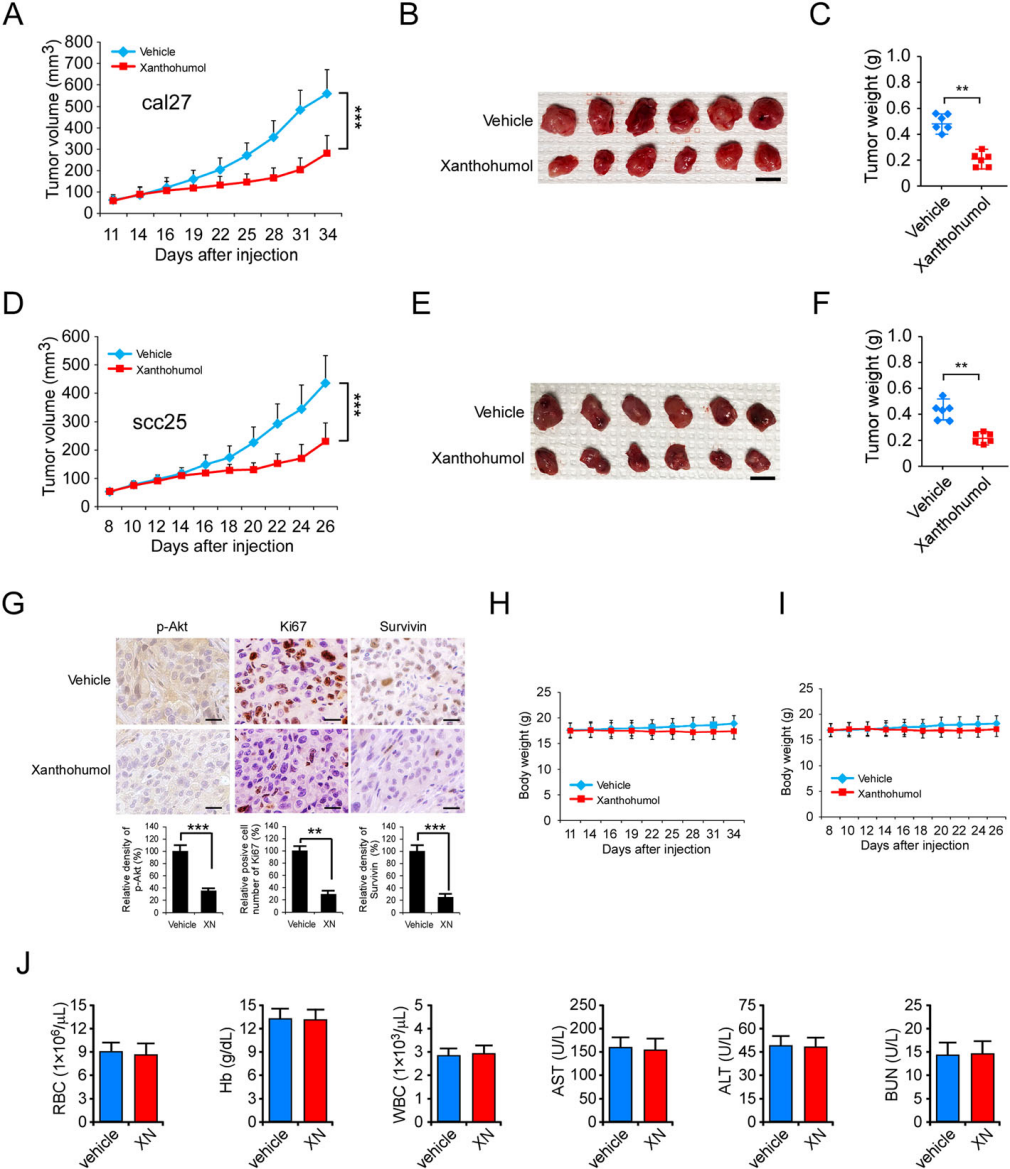
Xanthohumol suppresses in vivo xenograft tumor growth. **a**-**c**, Xanthohumol inhibited CAL27-derived xenograft tumor growth. The tumor volume (**a**), the image of tumor mass (**b**), and tumor weight (**c**) of vehicle- and xanthohumol-treated tumors. **d**-**f** Xanthohumol inhibited SCC25-derived xenograft tumor growth. The tumor volume (**d**), image of tumor mass (**e**), and tumor weight (**f**) of vehicle- and xanthohumol-treated tumors. For **b** and **e**, Scale bar, 1 cm. **g** IHC staining analysis of Ki67, p-Akt, and survivin in SCC25-derived xenograft tumors. Scale bar, 25 μm. **h** and **i** The body weights of CAL27 (**h**) and SCC25 (**i**) tumor-bearing mice with vehicle or xanthohumol treatment. **j** Blood analysis of mice with vehicle or xanthohumol treatment. **p < 0.01, ***p < 0.001

To determine the in vivo anti-tumor efficacy of xanthohumol, we performed the xenograft mouse model. Our data showed that xanthohumol significantly delayed in vivo tumor development of CAL27- and SCC25-derived xenograft tumors (Fig. 6a-f). Treatment with xanthohumol reduced the tumor volume (Fig. 6a and b) and tumor weight (Fig. 6c) of CAL27 tumors, the tumor volume of the vehicle-treated group reached at around 600 mm^3^, whereas the tumor volume of xanthohumol-treated group was less than 200 mm^3^ (Fig. 6a). A similar inhibitory effect was also observed in SCC25 xenograft tumors, and the tumor size of xanthohumol-treated group was significantly smaller than that of vehicle-treated group (Fig. 6d). The IHC data revealed that xanthohumol-treated xenograft tumors exhibited a significant reduction of Ki67, p-Akt, and survivin protein levels (Fig. 6g).

To determine the in vivo toxicity of xanthohumol, we recorded the bodyweight of xanthohumol-treated mice. We found that xanthohumol did not cause a significant body weight loss (Fig. 6h and i). Moreover, blood analysis showed that xanthohumol did not affect the RBC and WBC counts. In addition, xanthohumol exhibited no significant toxicity to vital organ functions of bone marrow, kidney, and liver, as the Hb, AST, ALT, and BUN in vehicle- and xanthohumol-treated mice was unaffected (Fig. 6j). Overall, these data suggest that xanthohumol is a well-tolerated natural compound which suppresses in vivo tumor growth of OSCC cells.

### Xanthohumol overcomes radioresistance in OSCC cells

**Fig. 7:**
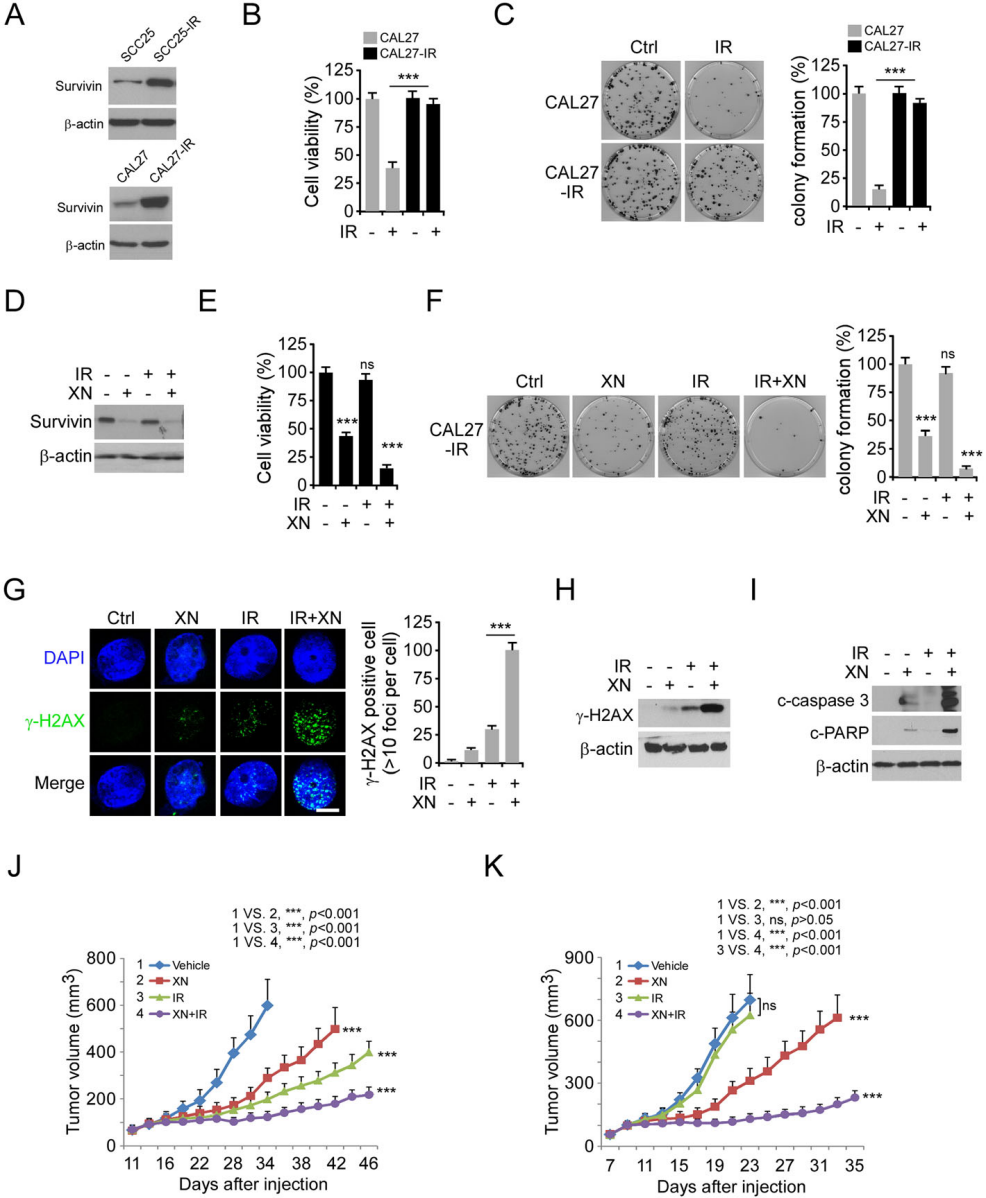
Xanthohumol overcomes radioresistance in OSCC cells. **a** IB analysis of the expression of survivin in CAL27/CAL27-IR and SCC25/SCC25-IR cells. **b** The effect of irradiation (IR) on cell viability of CAL27/CAL27-IR cells. CAL27 and CAL27-IR cells were treated with 4 Gy IR, cell viability was examined 72 h later by MTS assay. **c** The effect of IR on colony formation of CAL27/CAL27-IR cells. CAL27 and CAL27-IR cells were treated with 4 Gy IR, colony number was examined 2 weeks later. **d** IB analysis of survivin protein level in CAL27-IR cells treated with xanthohumol (5 μM), IR (4 Gy), or a xanthohumol + IR combination. **e** and **f** The cell viability (**e**) and colony formation (**f**) of CAL27-IR cells treated with xanthohumol, IR, or a xanthohumol + IR combination. **g** and **h** Immunofluorescence (**g**) or IB (**h**) analysis of γ-H2AX in CAL27-IR cells treated with xanthohumol, IR, or a xanthohumol + IR combination. Scale bar, 5 μm. **i** IB analysis of apoptosis in CAL27-IR cells treated with xanthohumol, IR, or a xanthohumol + IR combination. **j** In vivo tumorigenesis of CAL27 cells treated with vehicle control, xanthohumol, IR, or a xanthohumol + IR combination. **k** In vivo tumorigenesis of CAL27-IR cells treated with vehicle control, xanthohumol, IR, or a xanthohumol + IR combination. ***p < 0.001. ns, not statistically significant

To determine the crucial role of survivin in radioresistance, we examined the protein level of survivin in two pair of radioresistant OSCC cells, CAL27/CAL27-IR and SCC25/SCC25IR. The IB data showed that survivin is highly expressed in both radioresistant CAL27-IR and SCC25-IR cells (Fig. 7a). Irradiation (4 Gy) significantly decreased the cell viability and colony formation of CAL27 and SCC25, but not that of the radioresistant cells (Fig. 7b and c, Supplementary Figure 3A and B). Importantly, treatment with xanthohumol reduced the expression of survivin in CAL27-IR and SCC25-IR cells robustly (Fig. 7d, Supplementary Figure 3C). In contrast, irradiation did not cause a significant decrease of survivin in radioresistant cells. Consistently, xanthohumol, but not single dose of irradiation, decreased the cell viability and colony formation of CAL27-IR and SCC25-IR cells (Fig. 7e and f, Supplementary Figure 3D and E). Furthermore, pretreated with xanthohumol significantly enhanced the anti-tumor effect of irradiation in these resistant cells (Fig. 7e and f, Supplementary Figure 3D and E). The IF and IB results revealed that treatment with xanthohumol facilitated irradiation-induced DNA damage, as the population of γ-H2AX positive cells was increased substantially (Fig. 7g and h). In addition, combination of xanthohumol with irradiation promoted apoptosis in CAL27-IR cells (Fig. 7i). To test whether xanthohumol overcomes radioresistance of OSCC cells in vivo, the CAL27 and CAL27-IR xenografts were treated with irradiation, xanthohumol, or in combination for 2 weeks. Xenografts derived from CAL27 cells were sensitive to irradiation with significant tumor shrinkage. In contrast, CAL27-IR xenografts were resistant to radiotherapy (Fig. 7j and k). Strikingly, xanthohumol delayed tumor development in both CAL27 and CAL27-IR derived xenografts, and the combination of xanthohumol sensitized CAL27-IR xenograft to radiotherapy (Fig. 7j and k), indicating that xanthohumol is able to overcome radioresistance of OSCC cells in vivo. A similar inhibitory effect was observed in SCC25 and SCC25-IR xenograft tumors, and the combination of xanthohumol with radiotherapy significantly suppressed the in vivo tumor growth (Supplementary Figure 3F and G). These results indicate that xanthohumol suppressed tumor growth and exhibited the potential to overcome radioresistance.

## Discussion

As the smallest member of the IAP family protein, survivin is frequently overexpressed in human cancers, including lung [24], prostate [25], colorectal [25], breast [26], ovarian [27], and liver [28] cancer. Previous studies have revealed that beyond regulation of cell division and mitosis, overexpression of survivin in cancer cells inhibits both extrinsic and intrinsic apoptosis signaling. Even the underlying mechanisms of survivin-mediated apoptosis suppression remains unclear, the interaction between survivin and the initiator or effector caspases directly or indirectly is considered to contribute to this process [10]. Importantly, the C-terminus of survivin is dispensable for cell division, whereas the N- terminus is required for apoptosis [29]. Moreover, a high level of survivin is related to enhanced angiogenesis, tumor invasion and metastasis, and chemo/radioresistance, and downregulation of survivin reversed these functions [25, 30, 31]. For example, overexpression of survivin confers insulin-like growth factor-induced lapatinib resistance in head and neck squamous carcinoma cells (HNSCC) [32]. Knockdown of survivin or pharmaceutical inhibition of survivin suppressed metastasis of ovarian cancer [27]. Furthermore, reduction of survivin expression by Brexpiprazole sensitizes glioma cells to osimertinib treatment [33]. In addition, suppression of survivin promotes the tumor-killing effect of radiotherapy in prostate cancer [34] and head and neck squamous cell carcinoma (HNSCC) [35, 36]. In the present study, we found that survivin is highly expressed in OSCC tumor tissues and cells, depletion of survivin expression by sgRNA blunted the malignant phenotypes in OSCC cells, including in vitro cell proliferation, colony formation, and in vivo tumor development. This evidence indicates that survivin is a promising target for OSCC clinical treatment in the future.

The expression of survivin in human cancer cells is tightly controlled at multiple levels, including transcription, translation, and posttranslational modification [10]. A panel of prooncogenic transcription factors, such as Sp1, NF-kB, STAT3, E2F1, and KLF5, have been demonstrated to bind with survivin promoter and enhance survivin expression in various tumors, whereas p53, forkhead box O3 (FOXO3), and early growth response 1 transcription factor (Egr-1) are negative regulators of survivin [10, 37]. Beyond transcriptional and translational regulation, recent reports revealed that survivin undergoes various posttranslational modifications, including phosphorylation, acetylation, and ubiquitination. Phosphorylation is required for survivin stabilization, subcellular trafficking, and biological activation. Several protein kinases have been demonstrated to phosphorylate survivin on multiple sites, such as Aurora-B kinase, polo-like kinase (Plk1), protein kinase A (PKA), cyclin-dependent kinase 1 (Cdk1), and casein kinase II (CKII) [10]. Phosphorylation on Thr34 is required for prevention of ubiquitination-induced survivin destruction [38]. Our data showed that the natural compound, xanthohumol, inhibited survivin Thr34 phosphorylation in an Akt-Weel-CDK1 signaling dependent manner. Mutation of Thr34 to Ala promoted survivin ubiquitination and reduced protein half-life, which may contribute to xanthohumol-mediated anti-tumor activity in vitro and in vivo. However, based on our current data, we could not exclude the possibility that xanthohumol reduced survivin phosphorylation on other serine or threonine sites. It would be interesting to figure out the downstream kinase targets of xanthohumol and identify other phosphorylation sites that are required for survivin stabilization.

Previous studies showed that the ubiquitin-proteasome pathway induces survivin degradation in a cell cycle-dependent manner [39]. So far, two E3 ligases, X-linked inhibitor of apoptosis (XIAP) and F-box Protein Fbxl7, have been identified to play key roles in the regulation of survivin stability. XIAP interacts with X-linked inhibitor of apoptosis (XIAP)-associated factor 1 (XAF1) and activates its E3 ligase activity to catalyze survivin ubiquitination and facilitates apoptosis [40]. Fbxl7 directly induces survivin ubiquitylation and degradation to orchestrate mitochondria function [22]. Furthermore, Aurora-A kinase promotes survivin stability through transcriptional and translational regulation of Fbxl7 expression in gastric cancer [41]. Exception of lysine-48 mediated survivin ubiquitination is required for degradation, lysine-63 linked polyubiquitination chains regulate chromosome alignment and segregation in mitosis. Lysine-63 de-ubiquitination of survivin by hFAM is required for the dissociation of survivin from centromeres. In contrast, ubiquitin-binding protein Ufd1 promoted lysine-63 ubiquitination is required for the association of survivin with centromeres [42]. Importantly, the deubiquitinase, such as CSN5/Jab1, which removes the K-48 linked ubiquitination chains from survivin, stabilized survivin and promotes non-small cell lung cancer cell growth [43]. Likewise, the ubiquitin-like protein FAT10 promotes bladder cancer progression by deubiquitination and stabilization of survivin[44]. Our data showed that the natural compound, xanthohumol, reduced survivin protein level by enhancing Fbxl7-mediated ubiquitination and degradation. Xanthohumol promoted the interaction between Fbxl7 and survivin, which in turn facilitated Fbxl7-mediated survivin destruction and decreased survivin expression in OSCC cells. This mechanism is different from the current survivin suppressant YM155. YM155 robustly inhibits survivin activity via disruption of Sp1-DNA interaction in the survivin core promoter, thus inhibits survivin transcription [45], whereas xanthohumol is a potential survivin inhibitor which regulates survivin expression through posttranslational modification. Our data suggest that enhancement of the E3 ligase-mediated ubiquitination and reduction of protein stability is a promising anti-tumor strategy for cancer treatment, and xanthohumol exhibited the potential to be used as a proteolysis targeting chimeras (PROTACs) [46] for novel therapeutic modality in drug discovery.

Xanthohumol is one of the most abundant prenylated flavonoids in hops (*Humulus lupulus* L.). Pharmacology studies showed that xanthohumol exhibits multiple biological functions, including anti-inflammatory, antioxidant, antibacterial, and antiviral activity [47]. Recently, accumulating evidence indicates that xanthohumol possesses the potent anti-cancer efficacy in various tumor models, such as hepatocellular carcinoma, leukemia, melanoma, non-small cell lung cancer, colorectal, ovarian, pancreatic, and cervical cancer [48]. Mechanistic studies revealed that induction of cell cycle arrest, inhibition of glycolysis, promotion of DNA damage and apoptosis, and suppression of angiogenesis/metastasis contribute to the anti-tumor activity of xanthohumol [48–50]. Beyond that, the combination of xanthohumol with other therapeutic agents enhanced the tumor-killing effect of chemotherapy in various tumor models [51–53]. In this study, we unexpectedly discovered that xanthohumol promoted survivin ubiquitination and degradation, which is required for xanthohumol-mediated tumor suppression in OSCC cells. Importantly, in combination with radiation, xanthohumol overcomes radioresistance in OSCC xenograft tumors. These findings extend our understanding of the anti-tumor mechanisms of xanthohumol and offer a novel alternative opportunity for cancer treatment.

## Conclusion

In summary, we identify that xanthohumol inhibits survivin phosphorylation by deregulation of Akt-Wee1-CDK1 signaling and eventually promotes survivin ubiquitination and destruction by E3 ligase Fbxl7. Thus, targeting this oncoprotein for degradation might be a promising strategy for anti-tumor therapy.

## Supplementary information

**Additional file 1: Table S1.** Screened compound list.

**Additional file 2: Figure S1.** A, Ectopic overexpression of survivin compromised xanthohumol-induced cell viability reduction. CAL27 cells were transfected with survivin cDNA and treated with xanthohumol for 24, cell viability was determined by MTS assay. B, CAL27 cells were treated as in “Supplementary Figure 1A”, whole-cell lysate was subjected to cleaved-caspase 3 activity analysis. C, CAL27 cells were treated as in “Supplementary Figure 1A”, whole-cell lysate was subjected to IB analysis. H, CAL27 cells were treated as in “Supplementary Figure 1A”, subcellular fractions were isolated and subjected to IB analysis. ***p < 0.001.

**Additional file 3: Figure S2.** The effect of xanthohumol on survivin transcription. OSCC cells were treated with xanthohumol for 24 h followed by the qRT-PCR analysis of survivin mRNA level. ns, not statistically significant.

**Additional file 4: Figure S3.** Xanthohumol overcomes radioresistance in OSCC cells. A, The effect of irradiation (IR) on cell viability of SCC25/SCC25-IR cells. SCC25 and SCC25-IR cells were treated with 4 Gy IR, cell viability was examined 72 h later by MTS assay. B, The effect of IR on colony formation of SCC25/SCC25-IR cells. SCC25 and SCC25-IR cells were treated with 4 Gy IR, colony number was examined 2 weeks later. C, IB analysis of survivin protein level in SCC25-IR cells treated with xanthohumol (5 μM), IR (4 Gy), or a xanthohumol + IR combination. D and E, The cell viability (D) and colony formation (E) of SCC25-IR cells treated with xanthohumol, IR, or a xanthohumol + IR combination. ***p < 0.001. F, In vivo tumorigenesis of SCC25 cells treated with vehicle control, xanthohumol, IR, or a xanthohumol + IR combination. G, In vivo tumorigenesis of SCC25-IR cells treated with vehicle control, xanthohumol, IR, or a xanthohumol + IR combination. ***p < 0.001. ns, not statistically significant.

## Data Availability

Materials are available upon request.

## Abbreviations

OSCC: Oral squamous cell carcinoma
XN: Xanthohumol
CPC: Chromosomal passenger complex
IAPs: Inhibitor of apoptosis protein family
HNSCC: Head and neck squamous cell carcinoma
FOXO3: Forkhead box O3
Egr-1: Early growth response 1 transcription factor
Plk1: Polo-like kinase
PKA: Protein kinase A
Cdk1: Cyclin-dependent kinase 1
CKII: Casein kinase II
XIAP: X-linked inhibitor of apoptosis
XAF1: X-linked inhibitor of apoptosis (XIAP)-associated factor 1
IB: Immunoblotting
IHC: Immunohistochemical staining
CHX: Cycloheximide
Cyto: Cytoplasmic fraction
Mito: Mitochondrial fraction
RBC: Red blood cells
WBC: White blood cells
Hb: Hemoglobin
ALT: Alanine aminotransferase
AST: Aspartate aminotransferase
BUN: Blood urea nitrogen
