# Supplementary figures and images for: Promotion of ubiquitination-dependent survivin destruction contributes to xanthohumol-mediated tumor suppression and overcomes radioresistance in human oral squamous cell carcinoma
Source: J Exp Clin Cancer Res. 2020 May 14;39:88. doi: 10.1186/s13046-020-01593-z (PMC7227341; doi:10.1186/s13046-020-01593-z)

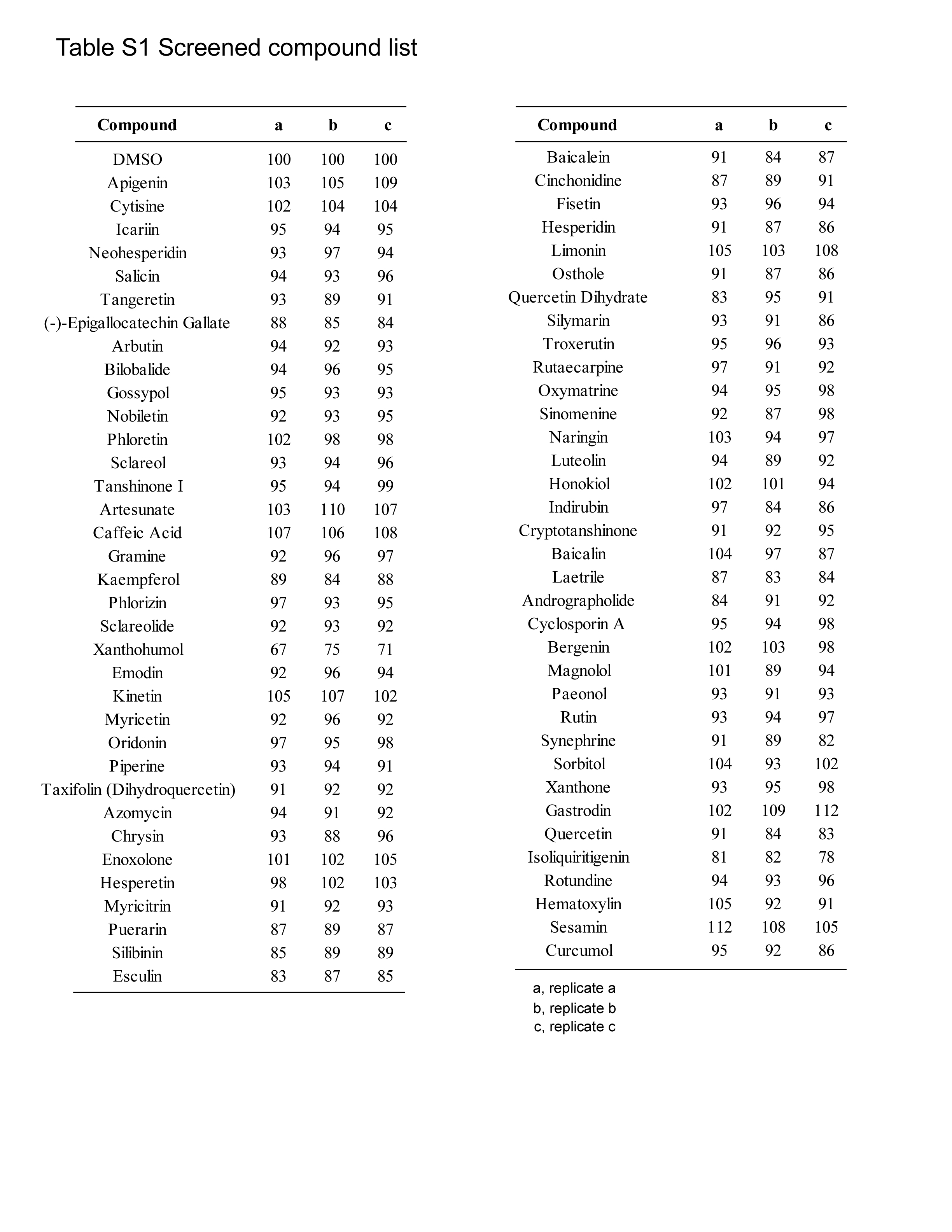

Supplement: Supplementary file 1 — Additional file 1: Table S1. Screened compound list. [file 13046_2020_1593_MOESM1_ESM.jpg]

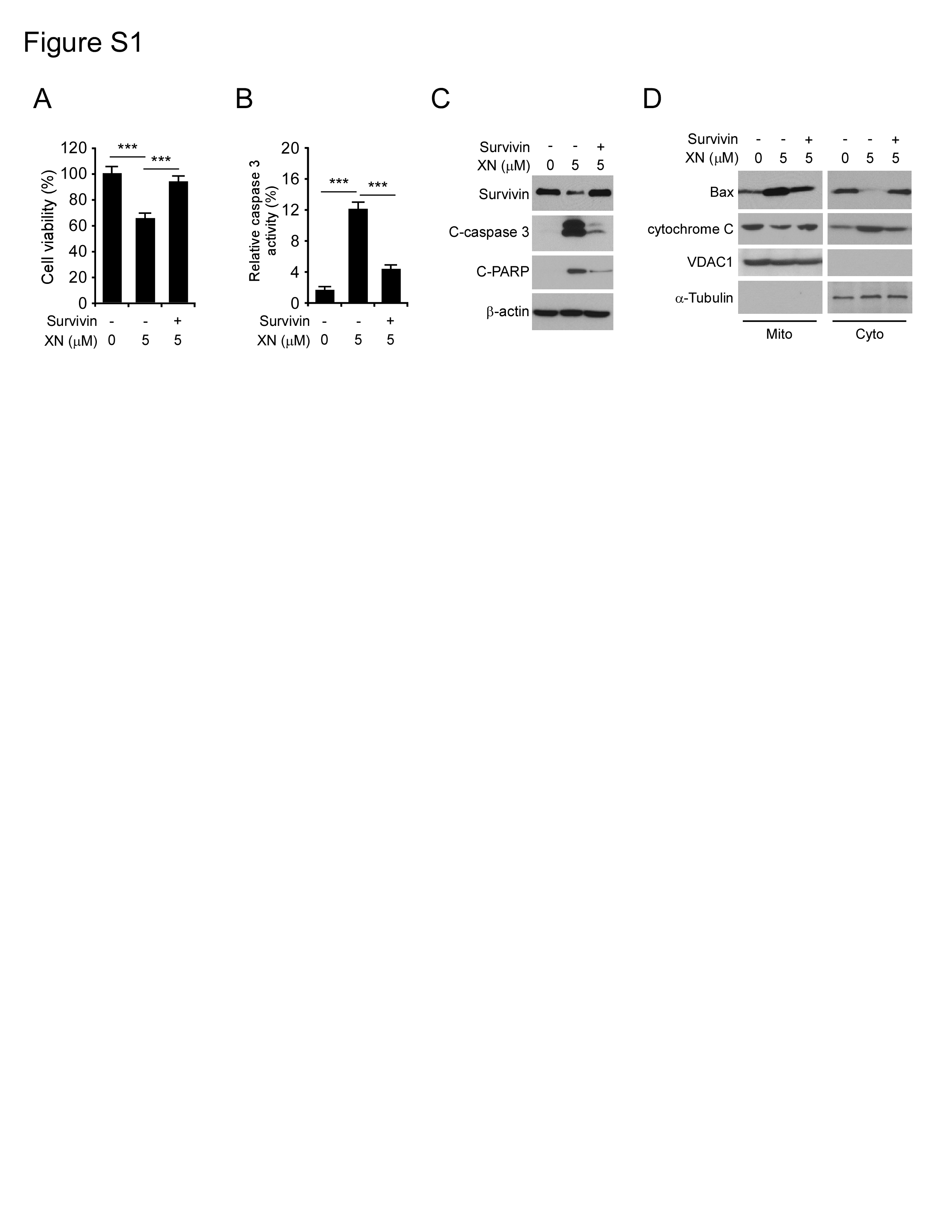

Supplement: Supplementary file 2 — Additional file 2: Figure S1. A, Ectopic overexpression of survivin compromised xanthohumol-induced cell viability reduction. CAL27 cells were transfected with survivin cDNA and treated with xanthohumol for 24, cell viability was determined by MTS assay. B, CAL27 cells were treated as in “Supplementary Figure 1A”, whole-cell lysate was subjected to cleaved-caspase 3 activity analysis. C, CAL27 cells were treated as in “Supplementary Figure 1A”, whole-cell lysate was subjected to IB analysis. H, CAL27 cells were treated as in “Supplementary Figure 1A”, subcellular fractions were isolated and subjected to IB analysis. ***p < 0.001. [file 13046_2020_1593_MOESM2_ESM.jpg]

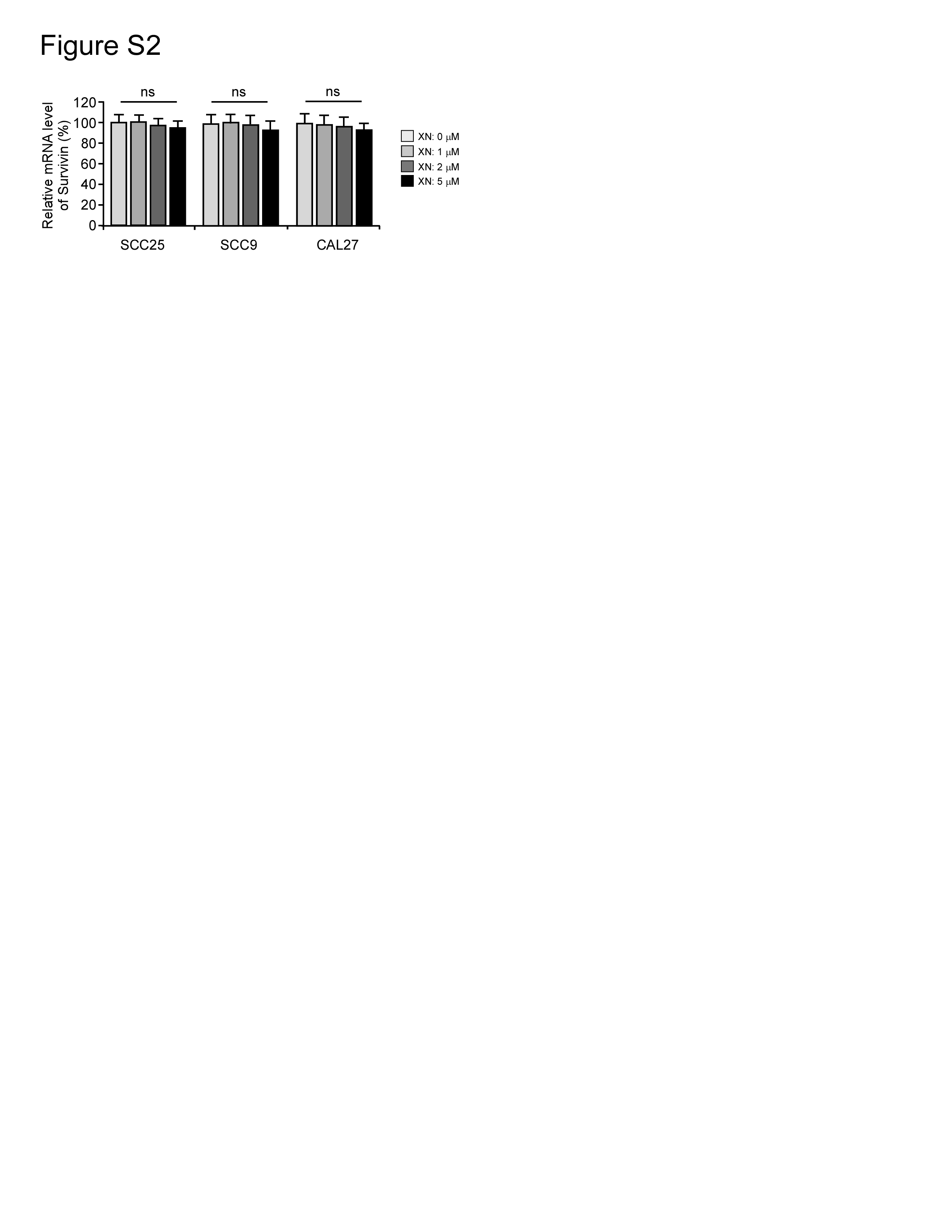

Supplement: Supplementary file 3 — Additional file 3: Figure S2. The effect of xanthohumol on survivin transcription. OSCC cells were treated with xanthohumol for 24 h followed by the qRT-PCR analysis of survivin mRNA level. ns, not statistically significant. [file 13046_2020_1593_MOESM3_ESM.jpg]

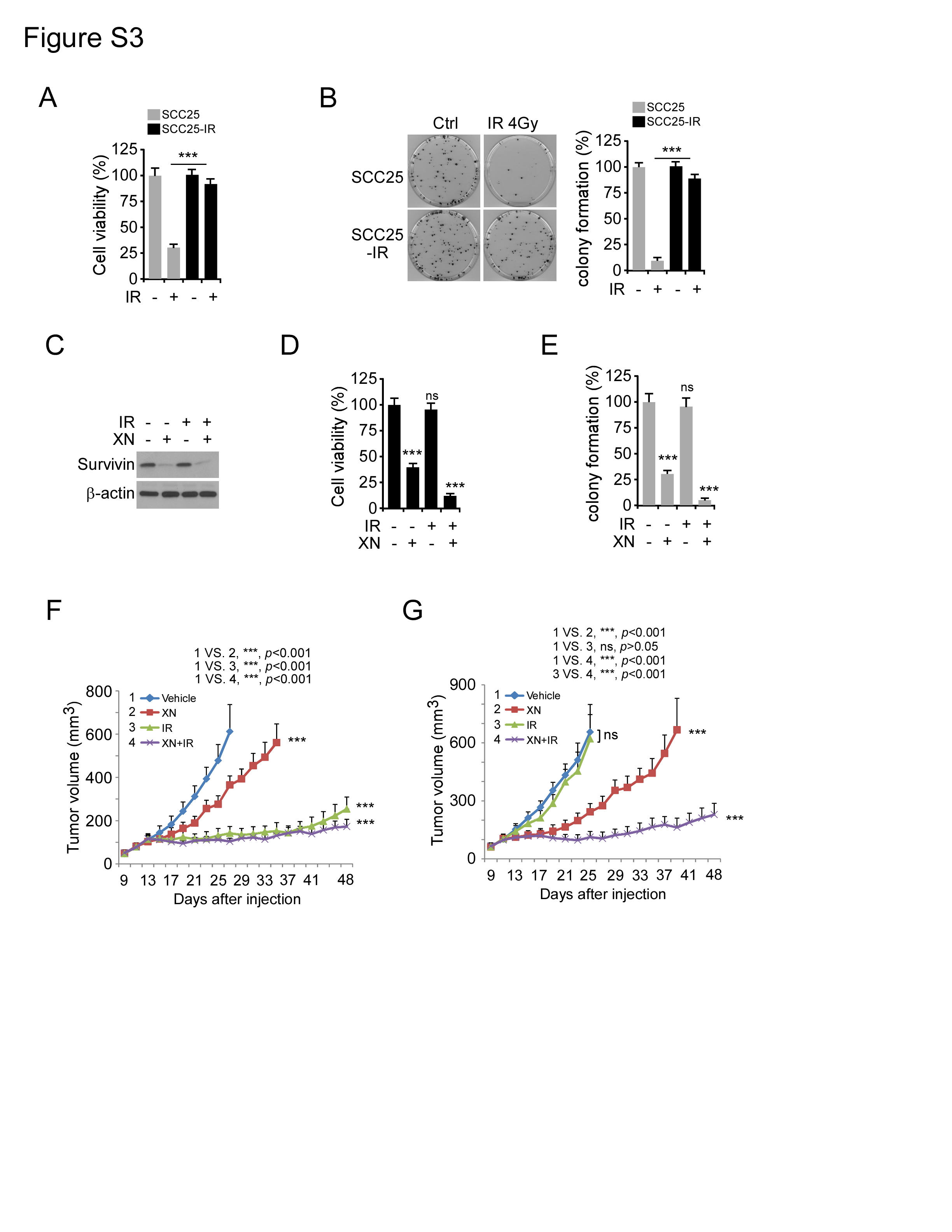

Supplement: Supplementary file 4 — Additional file 4: Figure S3. Xanthohumol overcomes radioresistance in OSCC cells. A, The effect of irradiation (IR) on cell viability of SCC25/SCC25-IR cells. SCC25 and SCC25-IR cells were treated with 4 Gy IR, cell viability was examined 72 h later by MTS assay. B, The effect of IR on colony formation of SCC25/SCC25-IR cells. SCC25 and SCC25-IR cells were treated with 4 Gy IR, colony number was examined 2 weeks later. C, IB analysis of survivin protein level in SCC25-IR cells treated with xanthohumol (5 μM), IR (4 Gy), or a xanthohumol + IR combination. D and E, The cell viability (D) and colony formation (E) of SCC25-IR cells treated with xanthohumol, IR, or a xanthohumol + IR combination. ***p < 0.001. F, In vivo tumorigenesis of SCC25 cells treated with vehicle control, xanthohumol, IR, or a xanthohumol + IR combination. G, In vivo tumorigenesis of SCC25-IR cells treated with vehicle control, xanthohumol, IR, or a xanthohumol + IR combination. ***p < 0.001. ns, not statistically significant. [file 13046_2020_1593_MOESM4_ESM.jpg]
